# Physiological adjustment effect of visual stimulation by fresh rose flowers on sympathetic nervous activity

**DOI:** 10.3389/fpsyg.2023.1159458

**Published:** 2023-04-27

**Authors:** Harumi Ikei, Chorong Song, Yoshifumi Miyazaki

**Affiliations:** ^1^Center for Environment, Health and Field Sciences, Chiba University, Kashiwa, Japan; ^2^Department of Forest Science, Kongju National University, Chungcheongnam-do, South Korea

**Keywords:** individual difference, law of initial value, roses, heart rate variability, sympathetic nervous activity, stress reduction, physiological adjustment effect, well-being

## Abstract

**Introduction:**

As modern societies are often stressful due to urbanization and artificialization, the physiological relaxing effects of natural environments or nature-derived stimuli on humans have attracted attention and scientific data are being accumulated. It is known that there is inter-individual variation in these effects. The study aim was to apply the law of initial values to investigate the physiological adjustment effect of viewing fresh roses on sympathetic nervous activity.

**Methods:**

In this crossover study, a total of 214 high school students, office workers, healthcare workers, and elderly people were analyzed. The participants viewed fresh roses in a vase for 4 min. In the control condition, participants did not view any fresh roses during the period. To offset any order effect, participants received visual stimuli in the order of fresh roses then the control (no fresh roses) or the control and then fresh roses. ln (LF/HF) of heart rate variability (HRV) obtained from a-a interval measurements using an acceleration plethysmograph and used as an index of sympathetic nervous activity. The initial value was ln (LF/HF) of HRV during the control viewing (no fresh roses), and the change value was ln(LF/HF) of HRV during visual stimulation by fresh roses minus the control viewing.

**Results and Discussion:**

The correlation between the two was assessed by determining Pearson’s correlation coefficient r, which was significantly negative. A physiological adjustment effect was observed such that participants with high initial sympathetic nervous activity showed a decrease in activity after visual stimulation with fresh roses, whereas participants with low initial activity showed an increase.

## 1. Introduction

Humans, which emerged 6–7 million years ago (Brunet et al., 2002), evolved in and became adapted to natural environments; thus, it is believed that the human body is built for natural environments (Miyazaki et al., 2011; O’Grady and Meinecke, 2015). This idea was proposed by Yoshifumi Miyazaki, the founder of nature therapy research, as the “back to nature” theory (Miyazaki, 2018). Humans have spent more than 99.99% of our evolutionary history in natural environments. If we define the beginning of urbanization as the start of the Industrial Revolution, less than 0.01% of our history has been spent in modern artificial environments. The gap between the natural environment to which our physiology is best adapted and the highly urbanized and artificial environment in which we live contributes to the “stress state” experienced by modern humans (Nazarian and Lee, 2021; Ancora et al., 2022). The development and diffusion of computer and ICT technology has led to even more rapid changes. In 1984, American clinical psychologist Craig Brod defined a new type of stress termed “techno-stress” (Brod, 1984). Techno-stress has further aggravated stress levels among modern people (René, 2013).

In modern stressful societies, field and laboratory experiments have shown that humans physiologically relax when exposed to various types of nature-derived stimuli. In field experiments, walking or viewing the surroundings in a forest or park has previously been shown to calm brain activity (Park et al., 2007; Song et al., 2020); increase parasympathetic nervous activity, which increases during relaxation (Kobayashi et al., 2015, 2018; Song et al., 2019a,b); decrease sympathetic nervous activity, which increases during stress (Kobayashi et al., 2015, 2018; Song et al., 2019a,b); and decrease stress hormone (cortisol) levels (Park et al., 2010; Kobayashi et al., 2017). In laboratory experiments, viewing flowers or foliage plants, smelling flowers or natural essential oils, listening to the sound of a babbling brook, and touching wooden plates have been shown to calm brain activity (visual stimuli: Song et al., 2017, 2018; Nakamura et al., 2019; Ikei et al., 2020; olfactory stimuli: Igarashi et al., 2014b; Ikei et al., 2015a,b; auditory stimuli: Jo et al., 2019; tactile stimuli: Ikei et al., 2017a,b, 2018a,b, 2019; Ikei and Miyazaki, 2020); increase parasympathetic nervous activity, which increases during relaxation (visual stimuli: Ikei et al., 2013, 2014a,b, 2020; Komatsu et al., 2013; Jo et al., 2022; olfactory stimuli: Igarashi et al., 2014a; Ikei et al., 2015a, 2016; Joung et al., 2014; tactile stimuli: Ikei et al., 2017a,b, 2018a,b, 2019; Ikei and Miyazaki, 2020); and decrease sympathetic nervous activity, which increases during stress (visual stimuli: Ikei et al., 2013, 2014b, 2020; Igarashi et al., 2015, auditory stimuli: Jo et al., 2019; tactile stimuli: Ikei et al., 2018b, 2019; Ikei and Miyazaki, 2020). As mentioned above, exposure to various types of nature has been shown to have a physiologically relaxing effect on subjects.

In developed countries, as people typically spend more than 80% of their time indoors (Leech et al., 2002), their well-being and quality of life are greatly influenced by the indoor environment. There is growing interest in the stress-relieving effects of flowering plants and houseplants, which are commonly used to decorate homes and offices as small natural features that can be incorporated into daily life (Jo et al., 2019). *Rosa* (Family *Rosaceae*) species, commonly known as rose, have long been popular as ornamental and medicinal plants (Krussman, 1981). Today, roses are one of the most commonly cultivated plants in the world (Gudin, 2003) and have been empirically shown to produce relaxing effects in humans. For example, Ikei et al. (2014a) conducted a crossover study involving male office workers to investigate the effects of brief visual stimulation with fresh roses on autonomic nervous activity. The participants observed 30 unscented pink roses placed in a cylindrical glass vase for 4 min. The results showed that visual stimulation with fresh roses significantly increased high frequency (HF) of heart rate variability (HRV), which reflects parasympathetic nervous activity, compared to the control condition where no roses were viewed. A crossover study using a similar experimental protocol conducted with male and female high school students (Ikei et al., 2013) and female healthcare workers (Komatsu et al., 2013) also found that visual stimulation with fresh roses significantly increased HF of HRV (Ikei et al., 2013), significantly decreased ratio of low frequency to high frequency (LF/HF) of HRV, a measure of sympathetic nervous activity (Ikei et al., 2013), and pulse rate (Komatsu et al., 2013). Other studies have reported that visual stimulation with unscented red roses leads to a calming effect on prefrontal cortex activity (Song et al., 2017) and that visual stimulation with different petal colors produces different physiological responses (Xie et al., 2021).

While conducting these studies, we discovered an interesting phenomenon (Song et al., 2015). In forest therapy-induced changes in blood pressure, the phenomenon, which was named the “physiological adjustment effect,” was found to decrease blood pressure in subjects with high blood pressure and increase blood pressure in subjects with low blood pressure. However, there have been no reports examining the physiological adjustment effect of fresh rose flowers, a form of nature that is familiar in the daily lives of modern people.

The present study aim was to measure the sympathetic nervous activity of 214 participants while they viewed fresh roses, with the goal of characterizing their physiological adjustment effects.

## 2. Materials and methods

We previously reported the physiological relaxation effects of visual stimulation with fresh roses in high school students (Ikei et al., 2013), office workers (Ikei et al., 2014a), and healthcare workers (Komatsu et al., 2013). The present study also considered elderly people (*n* = 100), for a total of 214 participants (Table 1). The results are presented with a focus on the “physiological adjustment effect.” This study was conducted in accordance with the guidelines of the Declaration of Helsinki, and the protocol was approved by the Ethics Committee of the Center for Environment, Health and Field Sciences, Chiba University (project identification number 5).

**Table 1:** Participants’ demographic characteristics.

Parameter	Total	High school students	Office workers	Healthcare workers	Elderly persons
Number	214 (M: 117, F: 97)	55 (M: 36, F: 19)	45 (M: 31, F: 14)	14 (M: 0, F: 14)	100 (M: 50, F: 50)
Age (years)	46.6 ± 23.0	15.5 ± 0.5	37.8 ± 10.5	42.1 ± 12.6	68.2 ± 4.2
Height (cm)	163.1 ± 9.1	166.5 ± 8.5	166.7 ± 7.8	*No data	159.7 ± 8.6
Weight (kg)	58.6 ± 11.0	55.2 ± 9.0	62.4 ± 12.6	*No data	58.7 ± 10.8

Mean ± standard deviation, M: Male, F: Female, *No data for healthcare workers because their heights and weights had not been collected.

The visual stimuli were pink, scentless, fresh roses (Dekora) cut into 40 cm lengths and arranged in cylindrical glass vases (12 cm diameter, 20 cm high), 37 to 40 cm away from the participant’s eyes. The control was no fresh roses.

An accelerated plethysmograph (Artet C, Umedica Inc., Osaka, Japan, Takada et al., 2004) was used to evaluate sympathetic nervous activity due to heart rate variability (HRV). HRV is the fluctuation in the time interval between heart beats and is widely used as an objective assessment of stress-relaxation states (Kim et al., 2019). Pulse waves were continuously measured by placing each participant’s left index finger on the sensor. The obtained a–a interval time series data were analyzed by the maximum entropy method and high frequency (HF, 0.15–0.40 Hz) and low frequency (LF, 0.04–0.15 Hz) power values were calculated (Kanaya et al., 2003). The LF/HF ratio reflects sympathetic nervous activity (Pagani et al., 1986; Camm et al., 1996), which increases during arousal and stress states (Järvelin-Pasanen et al., 2018; Thielmann et al., 2021) and decreases during drowsy states (Alexandru et al., 2021). LF/HF data were converted to natural logarithms for normalization (Kobayashi et al., 2012).

**Figure 1:**
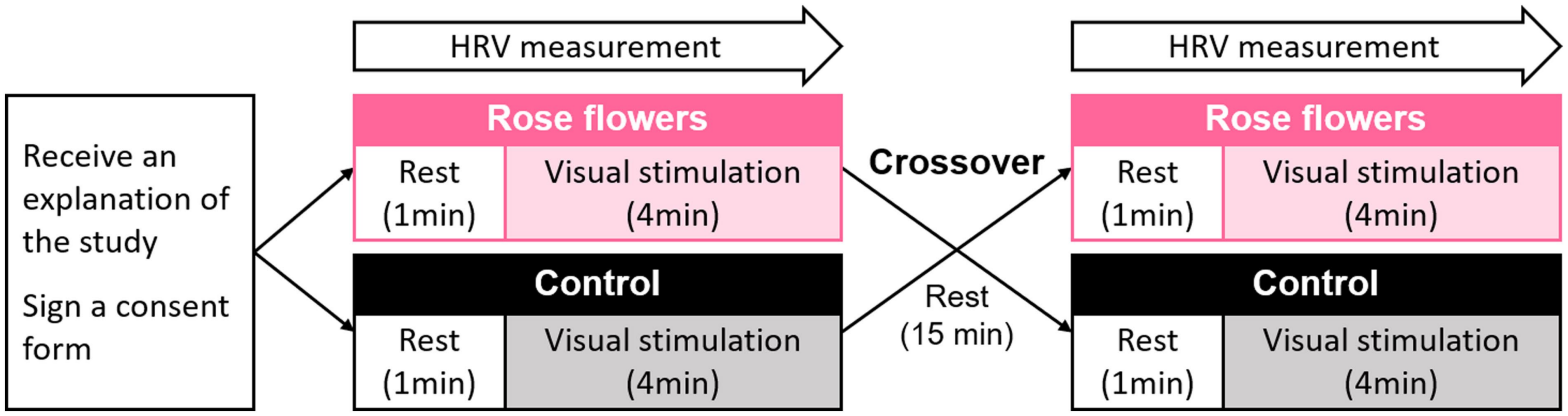
Design of the crossover study to measure the physiological adjustment effect of visual stimulation by fresh roses.

**Figure 2:**
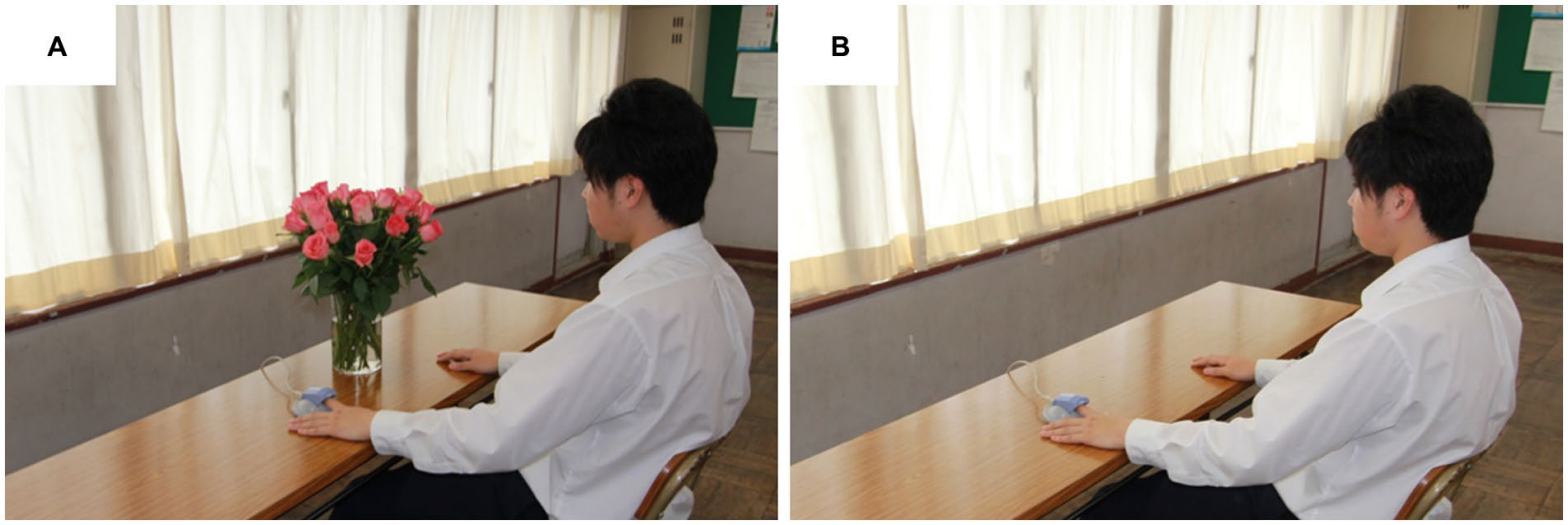
Experimental viewing conditions: **(A)** fresh roses condition and **(B)** control condition.

**Figure 3:**
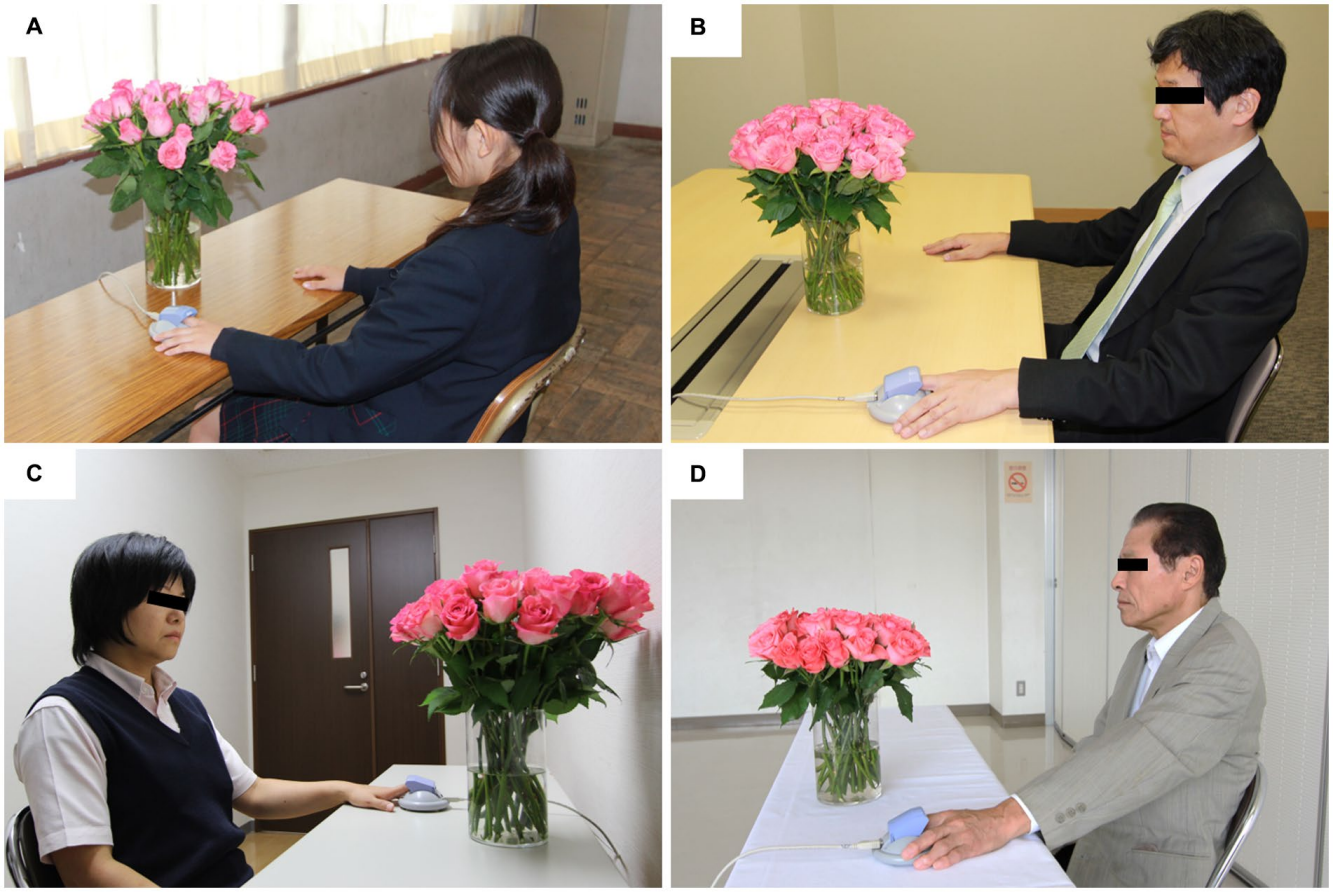
Measurement scene in the visual stimulation experiment of fresh rose flowers. **(A)** High school students, **(B)** office workers, **(C)** healthcare workers, **(D)** elderly persons.

The study protocol is presented in Figure 1. The participants entered the laboratory after receiving an explanation of the study and signing a consent form in the waiting room. After sitting and resting in a chair, the participants viewed fresh roses (Figure 2A) or the control (Figure 2B) for 4 min. Physiological responses were measured continuously from the beginning to the end of the resting period. After the end of the measurement, the effects of the order were counterbalanced by receiving a different stimulus from the first one. Figure 3 shows the experimental scene of visual stimulation with fresh roses for each attribute.

SPSS software version 28.0 (IBM SPSS Statistics for Windows, IBM Corp., Armonk, NY, United States) was used for statistical analysis. In addition to the new experimental data for elderly persons, we used data from the three previous experiments: high school students (Ikei et al., 2013), office workers (Ikei et al., 2014a), and healthcare workers (Komatsu et al., 2013). Initial values were defined as ln(LF/HF) of HRV during the control viewing (no fresh roses), and changes were defined as ln(LF/HF) of HRV during visual stimulation by fresh roses minus the ln(LF/HF) of HRV during the control viewing. Pearson’s correlation test was performed to determine the correlation coefficient *r* between the two. A value of *p* < 0.05 was considered to be indicative of statistical significance.

## 3. Results and discussion

**Figure 4:**
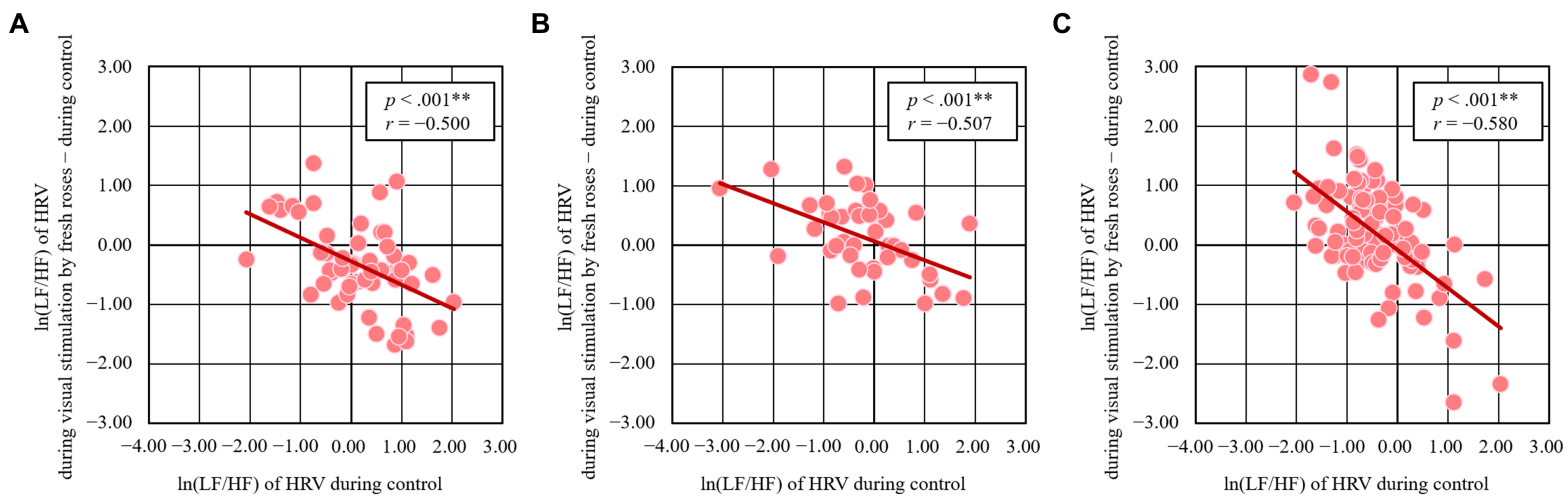
Relationship between the initial value and change in ln (LF/HF) of HRV induced by visual stimulation with fresh roses organized by group. **(A)** High school students, *N* = 55, ** *p* < 0.001 **(B)** office workers, *N* = 45, ** *p* < 0.001, and **(C)** elderly people. *N* = 100, **p < 0.001 by Pearson’s correlation.

The results for high school students (*N* = 55, 15.5 ± 0.5 years), office workers (N = 45, 37.8 ± 10.5 years), and elderly people (*N* = 100, 68.2 ± 4.2 years) with different attributes are shown in Figure 4; healthcare workers are excluded due to the small number of subjects. For these three groups, the initial (ln(LF/HF) of HRV during the control condition) and change (ln(LF/HF) of HRV during visual stimulation by fresh roses – control condition value) showed a significant negative correlation (high school students: *r* = −0.500, *p* < 0.001; office workers: *r* = −0.507, *p* < 0.001, elderly people: *r* = −0.580, *p* < 0.001).

**Figure 5:**
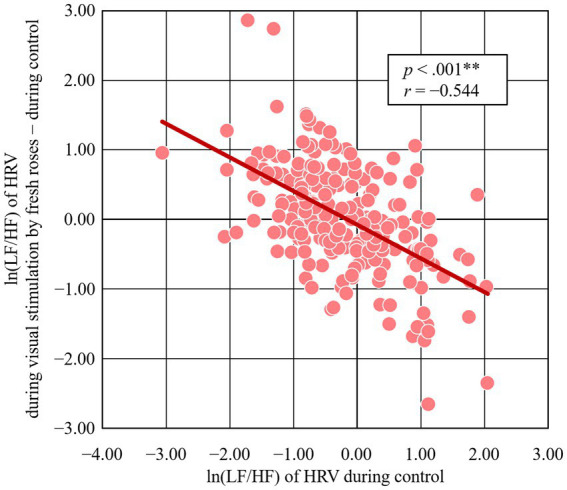
Relationship between the initial and change in ln (LF/HF) of HRV induced by visual stimulation with fresh roses. *N* = 214, ** *p* < 0.001 by Pearson’s correlation.

The relationship between the initial value and the change value in the ln(LF/HF) of HRV by visual stimulation of fresh roses in 214 participants is shown in Figure 5. There was a significant negative correlation (*r* = −0.544, *p* < 0.001) between the initial value (ln(LF/HF) of HRV during control) and changes (ln(LF/HF) of HRV during visual stimulation by fresh roses − during control).

Participants with high initial sympathetic nervous activity values showed a decrease in values upon visual stimulation with fresh roses, whereas participants with low initial values showed an increase in values upon visual stimulation. In previous studies, LF/HF of HRV has been reported to increase in awakening and stress states (Järvelin-Pasanen et al., 2018; Thielmann et al., 2021) and decrease in drowsy states (Alexandru et al., 2021). So far, nature therapy research has focused on the high LF/HF in the stress state that is decreased by nature-derived stimuli. The present study revealed that visual stimulation with fresh roses has a physiological adjustment effect, i.e., it not only decreases high LF/HF but also increases low LF/H in the drowsy state.

The law of initial values was proposed by Wilder in 1931 (Leites, 1936; Wilder, 1967) and has since been used in stressor studies in physiology. Lacey (1956) examined the relationship between initial values and change values in blood pressure and heart rate induced by stressors and reported that those with higher initial values were less responsive to stressors and other stimuli. Hord et al. (1964) found a significant correlation between initial values of heart rate and respiratory rate and changes induced by stressors.

Although previous studies in the law of initial values have focused on stressors that cause physiological stress states, recent studies have focused on the physiological relaxing effects of nature, such as forests. Song et al. (2015) examined changes in blood pressure and pulse rate in 92 Japanese male university students while they walked in a forest. They found a significant negative correlation between initial values and the amount of change. In other words, people with high initial values showed a decrease in blood pressure and pulse rate while walking in the forest, whereas people with low initial values showed an increase. These changes in blood pressure indicated that the changes in blood pressure approached the proper value, indicating that walking in the forest had a physiological adjustment effect. This physiological adjustment effect is unique to the forest environment because it was not observed when the same participants walked in the city. Yu and Hsieh (2020) found a significant negative correlation between the initial value and change in blood pressure in a 3-day forest therapy workshop for 23 Taiwanese aged 25–70 years.

The often stressful conditions found in modern artificial and urban environments encountered by humans have led to a growing interest in the physiological relaxing effects of nature, such as flowers, plants, and forests. In particular, the COVID-19 pandemic that began in 2019 and the resulting lockdowns and other behavioral restrictions have had significant negative effects on the daily lives of humans worldwide. We have previously reported that visual stimulation by fresh roses produced physiological relaxation effects in high school students (Ikei et al., 2013), office workers (Ikei et al., 2014a), and healthcare workers (Komatsu et al., 2013). The present study is the first to reveal the physiological adjustment effects of visual stimulation by fresh roses on sympathetic nervous activity in 214 participants, which included 100 elderly persons and a total of 114 other participants from the three earlier studies. The physiological adjustment effects of visual stimulation by fresh roses obtained by this study are expected to contribute to promoting and maintaining well-being in modern humans.

Future research efforts on this topic could include the following: (1) Although sympathetic nervous activity was used as a physiological index in this study, brain activity and endocrine activity should also be measured simultaneously to elucidate the mechanism of the physiological adjustment effect of roses. (2) Examinations of nature-derived stimuli other than roses and forests should be performed to elucidate the broader overall physiological adjustment effect of nature on humans.

## 4. Conclusion

In this study, high school students, office workers, healthcare workers, and elderly persons (214 participants in total) were examined for their sympathetic nervous activity upon viewing roses to determine its physiological adjustment effect. Participants with high initial sympathetic nervous activity showed a decrease in sympathetic nervous activity after visual stimulation with fresh roses, whereas participants with low initial activity showed an increase. Short-term visual stimulation with fresh roses had a beneficial physiological adjustment effect.

## Data availability statement

The data analyzed in this study is subject to the following licenses/restrictions: The datasets generated for this study are available on request to the corresponding author. Requests to access these datasets should be directed to Yoshifumi Miyazaki, ymiyazaki@faculty.chiba-u.jp.

## Ethics statement

The studies involving human participants were reviewed and approved by Ethics Committee of the Center for Environment, Health and Field Sciences, Chiba University, Japan. Written informed consent to participate in this study was provided by the participants themselves or by the participants’ legal guardian/next of kin if they were minors.

## Author contributions

YM: conceptualization, funding acquisition, project administration, and supervision. HI, CS, and YM: methodology, investigation, data curation, and writing—review and editing. HI: formal analysis and visualization. HI and YM: writing—original draft preparation. All authors have read and agreed to publish the final version of the manuscript.

## Funding

This work was supported by JSPS KAKENHI Grant Number JP22K13663 and Grant from the Policy Research Institute, Ministry of Agriculture, Forestry, and Fisheries in Japan, “Extramural Research Program for Agricultural, Forestry, and Fishery Policy Research.”

## Conflict of interest

The authors declare that this research was conducted in the absence of any commercial or financial relationships that could be construed as a potential conflict of interest.

## Publisher’s note

All claims expressed in this article are solely those of the authors and do not necessarily represent those of their affiliated organizations, or those of the publisher, the editors and the reviewers. Any product that may be evaluated in this article, or claim that may be made by its manufacturer, is not guaranteed or endorsed by the publisher.
